# Donor-intrinsic variables determine mobilization efficiency: analyses from a cohort of sixty twice-mobilized stem cell donors

**DOI:** 10.1186/s12967-020-02634-z

**Published:** 2020-12-18

**Authors:** Soo-Zin Kim-Wanner, Seo-Youn Lee, Erhard Seifried, Halvard Bonig

**Affiliations:** 1 German Red Cross Blood Service Baden-Wuerttemberg-Hessen, Institute Frankfurt, Frankfurt, Germany; 2 Institute for Transfusion Medicine, Goethe University Medical School, Haus 76, Sandhofstr. 1, 60528 Frankfurt, Germany; 3 Division of Hematology, Department of Medicine, University of Washington School of Medicine, Seattle, WA USA; 4 Present Address: Department of Regional Evaluation and Accounting, Hessian Cancer Registry, Office of State Examination and Examination in the Health Service, Frankfurt, Germany

**Keywords:** G-CSF, Mobilization, Stem cell, Allogeneic, Volunteer donor, CD34 + cells, Second donation

## Abstract

**Background:**

Healthy volunteer registry donors have become the backbone of stem cell transplantation programs. While most registrants will never become actual donors, a small minority are called upon twice, most commonly for the same patient because of poor graft function. Anecdotal evidence provides no hard reasons to disallow second-time mobilized apheresis, but few centers have treated enough two-time donors for definitive conclusions. Moreover, for reasons unknown, the efficiency of G-CSF varies greatly between donations.

**Methods:**

Comparison of outcomes of first vs. second donations can formally confirm G-CSF responsiveness as intrinsically, likely genetically, determined. In our database, we identified 60 donors (1.3%) who received two cycles of G-CSF 24 days to 4 years apart and systematically compared mobilization outcomes.

**Results:**

First and second mobilization and collection proceeded without severe or unusual adverse effects. First-time mobilization efficiency was highly predictive of second-time mobilization. Neither mobilization efficiency nor time lag between donations affected the similarity of first- and second-time mobilization outcomes.

**Conclusions:**

With the caveat that only donors with an unremarkable first donation were cleared for a second, our data indicate that a second donation is feasible, equally tolerable as a first donation, and efficient. Moreover, the data strongly support the notion of donor-intrinsic variables dictating mobilization response and argue against relevant damage to the stem cell compartment during mobilization with rhG-CSF.

## Background

Despite the recent shift towards haplo-identical sibling donation, allogeneic stem cell donation in the Western world with its small core families continues to rely on the unselfish donation of stem cells from healthy volunteer registry donors. Stem cells are extracted from bone marrow or after G-CSF-mobilization from peripheral blood using apheresis. Both constitute relevantly invasive medical interventions, so that for many years the community has been documenting short- and long-term donor outcomes in tens of thousands of donors to assess whether stem cell donation is unconditionally safe (provided the considerable list of contra-indications to stem and/or marrow donation are observed) and healthy volunteer donation thus medically and ethically acceptable [1–3]. To this day, there appears to be agreement, including on the part of the regulatory agencies, that careful donor evaluation and selection provided, stem cell donation from registry donors by either method is safe and therefore permissible. Because of the paucity of such occurrences, the acceptance of second-time mobilization of volunteer donors is, by contrast, largely based on assumptions and limited evidence [4–8]. Where available, a different donor will be selected for patients with post-transplant relapse; similarly, for a new patient with the same HLA type preferentially a different donor will be activated. This leaves the not very frequent events of brittle primary engraftment or impending secondary (not infrequently iatrogenic) graft failure as main indications for requests for second donations from the same donor. Stem cell mobilization in the allogeneic setting is induced with recombinant human G-CSF (rhG-CSF) which is administered, with minor local variations in the exact dosing and dosing schedule, subcutaneously for five days prior to apheresis on the fifth day, at least two hours after the most recent G-CSF injection. Despite very tight dosing schedules, mobilization efficiency is remarkably variable, in our hands by a factor of almost 100 between worst and best mobilizers. Sex and girth have been identified in some studies as modestly associated with mobilization efficiency [9]. These studies, the limited data from multiply mobilized donors, as well as data generated with different strains of mice, together are being seen as evidence of donor-intrinsic factors modifying G-CSF responsiveness. Extensive attempts at identifying these, for instance the study of certain polymorphisms in chemokine, cytokine or adhesion molecule genes, have, however, thus far not been yielding [10–13]. Analysis of second-time donors can support or reject the notion of donor-inherent G-CSF responsiveness. By virtue of a large donor database comprising over 4500 individual mobilization courses, we could extract donation data from 60 second-time donors and compare safety and efficacy of both mobilization cycles as well as assess effects of mobilization efficiency and time elapsed between donations on outcomes of the second mobilization. As we are showing, second-time mobilization is safe and similarly effective as the same donor’s first mobilization cycle.

## Material and methods

This is a retrospective study where second-time donors donating between 2009 and 2013 at the German Red Cross Blood Service Baden-Württemberg-Hessen, Institute Frankfurt, were identified in the donor database and data from both donations were extracted as well as the donor charts were individually reviewed. This pseudonymized retrospective analysis of routine clinical data is part of our JACIE mandated ongoing product quality review activity. The Ethics Committee of Goethe University Medical Center has confirmed that no informed consent or ethics review is required for these activities.

Methodology for donor assessment and clearance was previously reported [14]; the guidance of local and international guidelines was observed or exceeded. Mobilization was with rhG-CSF; nine donors from one of the registries our center serves received Lenograstim at a dose not exceeding 7.5 µg/kg day, all other 51 donors received Filgrastim at a dose not exceeding 10 µg/kg day, in both cases dosed to the nearest full syringe/vial, and administered as split-dose subcutaneous self-injection. Where the morning and evening doses differed, the higher dose was injected noctu. As we previously demonstrated, despite the minor per-kg-dose variation resulting from this scheme, no dose effect can be observed [15]. The first apheresis was started > 2 h after the ninth injection. The concentration of circulating “stem cells”, i.e. SSCmid-low/FSClow/CD45dim/CD34 + cells (referred to as CD34 + cells from hereon) [16], in blood collected immediately prior to the first apheresis was considered the “mobilization efficiency”. Donors undergoing a second independent mobilization and apheresis cycle were identified in the laboratory information system. Sixty donors were thus identified between 2009 and 2013. For these, G-CSF dose and circulating CD34 + count immediately preceding the apheresis were extracted (mobilization efficiency), as well as the date of the first apheresis in each apheresis cycle (to analyze potential effects of the inter-donation interval on second-time G-CSF responsiveness). Blood cell counts at work-up were extracted from the donor charts. Adverse events from mobilization were informally queried just prior to starting the apheresis. Except for the explicit questions “did you experience any bone pain, at what point during mobilization, did you take any acetaminophen for your bone pain”, other frequently observed non-severe adverse events were not specifically queried. Where no adverse events were recorded in the extracted data, absence of adverse events was assumed.

CD34 + cell enumeration was done by single-platform flow cytometry using the CE-marked SCE kit (Becton–Dickinson, Heidelberg, Germany) on the FACSCalibur with Cellquest software (Becton–Dickinson) [16]. Complete blood counts from donor blood and apheresis products were done with Sysmex XT1800 (Norderstedt, Germany).

Descriptive statistics, Pearson’s correlation coefficient between mobilization efficiency at the first and second donation as well as graphics were executed in excel for Microsoft 365 (Microsoft, Redmond, WA) and GraphPad Prism 5.0. Donations were grouped by mobilization efficiency (< 50/50–130/ > 130 CD34 + cells/µL) or by inter-donation interval (< 100/100–180/181–365/ > 365 days), the Delta (CD34 + cell concentration at time point 2 minus CD34 + cell concentration at time point 1) as well as the % [(CD34 + cell concentration at time point 2/CD34 + cell concentration at time point 1)-1] difference were calculated for each pair of donations, and the Wilcoxon signed rank test was used to test for possible effects of differential G-CSF responsiveness or time between donations. Calculations were done with the social sciences online statistics calculator https://www.socscistatistics.com/tests/signedranks/default.aspx.

## Results

Donor epidemiology: From 2009 to 2013 PBSC from 4579 allogeneic donors were collected at our institute; 1.3% (n = 60) presenting for a second donation. Consistent with a German registry population [17], donors were predominantly Caucasian, average age was 35 years, three-quarters (44/60) were male. Average weight, height and BMI were 80 kg, 179 cm, 25.0 kg/m^2^, i.e. half the donors were overweight or obese. Donor characteristics are shown in Table 1.

**Table 1:** Donor characteristics at first and second donation

Characteristic	1st donation			2nd donation		
	Female	Male	All	Female	Male	All
n	16	44	60	16	44	60
Median age (range)—years	31 (21–53)	32.5 (18–62)	32 (18–62)	31.5 (21–54)	34.5 (18–62)	33.5 (18–62)
Mean weight (range)—kg	71 (56–110)	83.5 (57–129)	80 (56–129)	71.3 (58–110)	84 (54–129)	81 (54–129)
Mean height (range)—cm	169 (160–180)	182 (169–192)	179 (160–192)	169 (160–180)	182 (170–192)	179 (160–192)
Mean CD34 (range)—/µl	75 (33.1–230.7)	96.9 (32.8–192.5)	91.1 (32.8–230.7)	84.6 (24.0–338.7)	83.9 (22.6–235.9)	84.1 (22.6–338.7)
Mean WBC (range)—10^3^/µl	6.9 (3.6–10.1)	6.1 (3.8–10.4)	6.3 (3.6–10.4)	6.9 (4.4–10.8)	5.9 (3.5–16.2)	6,1 (3.5–16.2)
Mean Hb (range)—g/L	136.9 (123–158)	153.3 (136–173)	14.9 (12.3–17.3)	132.6 (120–145)	153.1 (140–166)	14.8 (12–16.6)
Mean Hct (range)—%	39 (35.8–45.6)	43.5 (37.8–48.5)	42.3 (35.8–48.5)	37 (25.9–41.8)	43.5 (39.2–47.5)	41.8 (25.9–47.5)
Mean Plt (range)—10^3^/µl	263 (213–345)	229 (165–349)	238 (165–349)	274 (193–364)	224 (159–373)	237 (159–373)
Mean Lymphocyte (range)—10^3^/µl	1.9 (0.9–3.1)	1.7 (1.0–2.6)	1.7 (0.9–3.1)	1.8 (0.9–2.7)	1.5 (0.8–2.5)	1.6 (0.8–2.7)
Median duration time between donations—days				177 (35–659)	193 (24–1652)	187 (24–1652)

*WBC* white blood cell count, *Hb* hemoglobin, *Hct* hematocrit, *PLT* platelets, CD34 concentration is after G-CSF mobilization, blood counts are at donor work-up (unmobilized)

Mobilization and adverse events during mobilization: Mobilization was performed by self-injection of nine doses q12 hours as described in “Materials and methods”. Except for bone pain in almost all donors, adverse events were scarce and with the caveat that data quality on symptoms was sub optimal not discernibly different during the first and second round of G-CSF. We observed neither limiting, excessive or unusual adverse events during the second mobilization episode, therefore concluding about the safety of second mobilization of healthy donors The median time interval between first and second donation was 187 days (range 24–1652 days). No significant differences in platelets, hemoglobin and hematocrit were observed at work up between first and second-time point of apheresis, in agreement with published data [15]. Leucocyte and lymphocyte counts trended towards lower at second apheresis in male donors without statistically significance and remaining well within the range of normal.

**Fig. 1:**
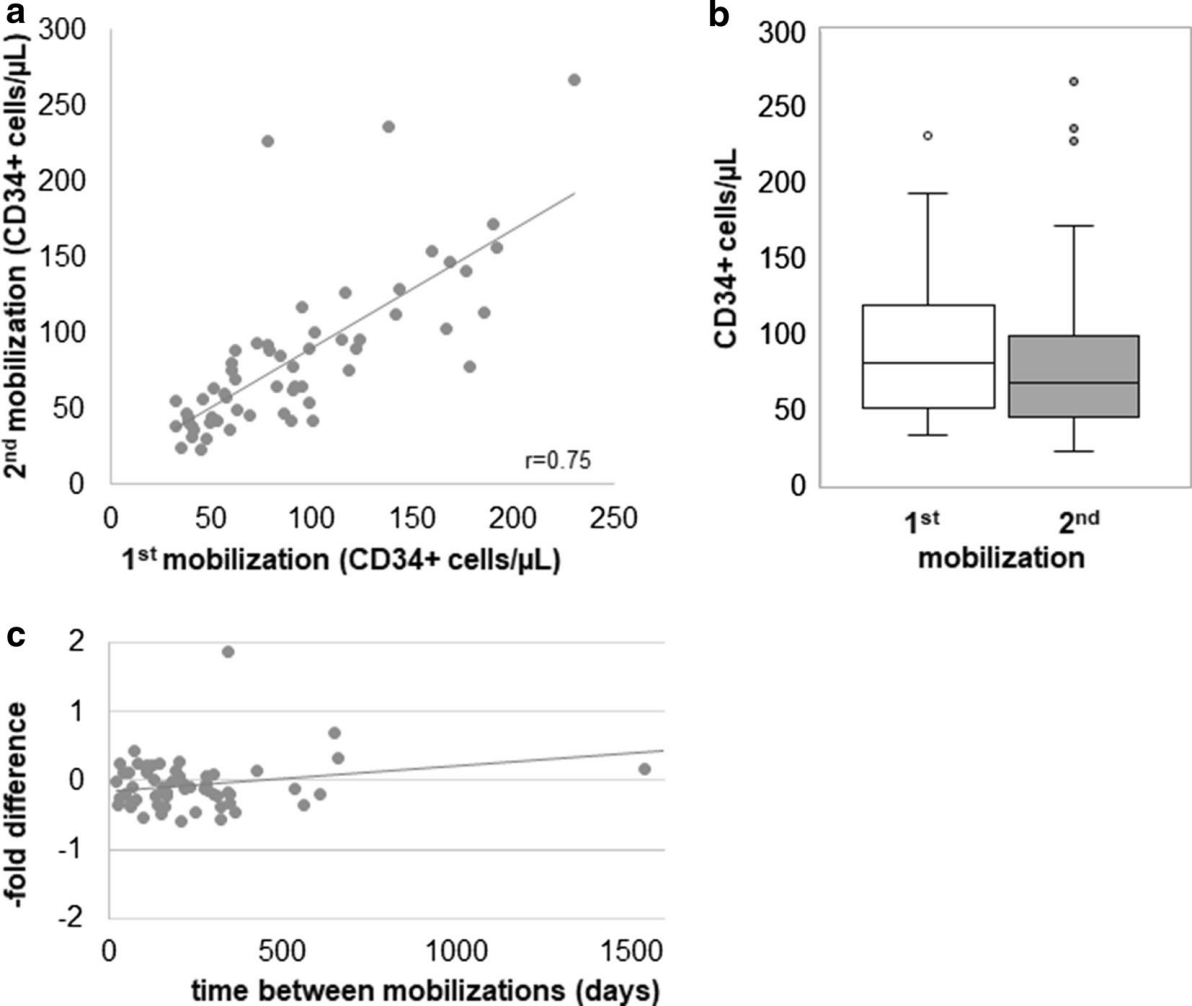
Correlation of first- and second-time mobilization. **a** Efficiency of first (X-axis) and second (Y-axis) mobilization of CD34 + cells are strongly correlated (Pearson correlation). The black line indicates the regression curve. Each dot represents one donor. **b** Efficiency of first and second mobilization of CD34 + cells (Y-axis) are shown in a box-whisker plot for comparison. No significant difference was detected. **c** Correlation of re-mobilization efficiency and time is shown as quotient (-fold difference) of second and first mobilization (Y-axis) over time between mobilization cycles (Y-axis). Each dot represents one donor (n.s.)

Mobilization efficiency: Range of mobilization was 23–267 CD34 + cells/µL (median: 77/µL); the mean Delta between first and second donation was − 8 CD34 + cells/µL or − 5%. 42% of the donations were within 10% of the mean of the two. In only 10% the difference in mobilization efficacy (in either direction) exceeded 50%. Mobilization efficiency after the first vs. second mobilization cycle was positively correlated with a Pearson's r = 0.75, p < 0.001 (Fig. 1a). The mean mobilization efficiency did not differ significantly between first and second donations (Fig. 1b), but we queried, whether the efficiency of the first mobilization response might affect that of the second. Given the slight deviation of the regression curve for first vs. second donation from the ideal correlation curve at higher mobilization yields, we specifically hypothesized that high mobilization responses might be relatively impaired during second apheresis whereas low or average mobilization was not. Therefore, the correlation of Delta or –fold difference over mobilization efficiency after the first mobilization was calculated but was very weak (not shown, r = 0.24). Furthermore, responses after the first cycle were categorized as relatively poor (n = 14, lowest quartile, < 50 CD34 + cells/µL), average (n = 34, 50–130 CD34 + cells/µL) or high (n = 12, > 130 CD34 + cells/µL) and comparison of first and second G-CSF responsiveness was compared separately by cohort. The mean Delta and % difference for category 1 was − 1 CD34 + cells/µL (1%), for category 2 − 6 CD34 + cells/µL (− 4%) and for category 3 − 27 CD34 + cells/µL (− 25%). A correlation between mobilization efficiency and similarity of mobilization responses, specifically relatively less efficient second mobilization of high mobilizers which the negative slope of the regression curve might seem to suggest, could not be detected (Mann–Whitney *U* test at the 0.05 level, not statistically significant). We next queried whether time between donations affected re-mobilization efficiency or more specifically, whether earlier re-mobilization might be relatively impaired. The correlation between time and Delta (absolute difference, i.e. mobilization after cycle 2 minus mobilization after cycle 1 in CD34 + cells/µL) or % difference were both very weak (r = 0.3), but the regression curves slanted modestly upward with increasing time (Fig. 1c). We therefore additionally generated categories of < 100 days/100–180 days/181–1 year/ > 1 year, incidentally approximately representing the quartiles in our cohort, and separately assessed differential mobilization in cycles one and two. Category 1 (n = 15) had a mean Delta of − 15 CD34 + cells/µL (− 11%), category 2 (n = 14) − 5 CD34 + cells/µL (− 9%), category 3 (n = 23) − 14 CD34 + cells/µL (− 7%) and category 4 (n = 8) + 14 CD34 + cells/µL (+ 18%), suggesting a trend towards slight impairment of mobilization at earlier time points and recovery beyond 1 year, but not reaching statistical significance at the 5% level, also not after only categorizing for < / > 1 year (not shown).

## Discussion

As we show in a sizeable cohort, re-mobilization of volunteer donors is safe, notably with the caveat that donors who experienced significant adverse events were not cleared for a second-time donation. These observations confirm earlier conclusions to this end by other groups [5, 18, 19]. Re-mobilization with G-CSF is also efficient. Specifically, we show that the second mobilization was similarly efficient as the first one. There was a trend to lower mobilization efficacy at second apheresis, diminishing over a period of time after one year, as was recently reported elsewhere [20]. Nevertheless a significant meaningful reduction of mobilization efficacy could not be seen at any time point as was similarly observed in another cohort [5, 6, 18, 19], whereas one earlier study had detected a significant reduction at the second donation, albeit in a smaller cohort of 30 [21]. Overall, and in line with previous observations, a high overall correlation between first and second apheresis is confirmed by this study with > 40% of donors showing almost identical mobilization efficacy at both donations irrespective of the time elapsed between donations [5, 19, 20]. Although a high variability in mobilization efficacy is generally acknowledged, the underlying mechanisms remain elusive. Besides associating factors like sex and weight, the high concordance in mobilization efficacy underscores a predominantly donor-inherent responsiveness [10, 22–24], at least in most of the donors, that might be genetically determined. Certain nucleotide polymorphism (SNP) have been controversially discussed to correlate with mobilization efficacy [11–13, 25, 26] and so far, definitive evidence for any imputable SNP is missing. The reason might be the lack of a sufficient number of samples to identify the responsible SNP(s) if present at low frequencies, confounded by presumably complex interaction of several positive and negative factors. Further studies, probably whole genome wide, are needed to clarify, whether and which genetic origin may be responsible for mobilization efficacy.

We observed neither limiting, excessive or unusual adverse events during the second mobilization episode, therefore concluding about the safety of second mobilization of healthy donors. The high concordance of mobilization efficacy in both donations may aid to predict product quality if a second donation is requested and guide donor management. Especially in donors with low mobilization efficacy a prediction of the expectable cell count in the product may affect choice of donor or stem cell source. Our study is single-center and, as a consequence of the rarity of the event, based on a moderately sized cohort. Moreover, side effects are not documented systematically and therefore conclusions have to be drawn carefully. Nevertheless, because of the homogeneity of the samples and analysis platform [16], our report adds to the body of data supporting safety, feasibility and effectiveness of second mobilized stem cell donation.

## Conclusion

This observation strongly underscores the prevalent notion that responsiveness to G-CSF is a donor variable, likely genetic, although the underlying genetics remain obscure. It also should be taken to indicate that mobilization with G-CSF does not relevantly deplete or otherwise functionally impair the stem cell compartment, an observation of relevance in light of emerging data about the high prevalence of clonal hematopoiesis (albeit by high resolution analysis only) even in young donors [27]. Effects of time on the efficiency of re-mobilization were, at best, very modest, and similarity of mobilization responses was observed irrespective of donors’ G-CSF responsiveness.

## Data Availability

Not applicable.

## Abbreviations

CD: Cluster of differentiation
G-CSF: Granulocyte colony-stimulating factor
Hb: Hemoglobin
Hct: Hematocrit
HLA: Human leukocyte antigen
JACIE: Joint Accreditation Committee of the International Society for Cell Therapy (ISCT) and European Bone Marrow Transplant Group (EBMT)
PLT: Platelets
rhG-CSF: Recombinant human G-CSF
SNP: Single nucleotide polymorphism
WBC: White blood cell count

## References

[1] Miller JP, Perry EH, Price TH, Bolan CD, Karanes C (2008). Recovery and safety profiles of marrow and PBSC donors: experience of the National Marrow Donor Program. Biol Blood Marrow Transpl J Am Soc Blood Marrow Transpl.

[2] Pulsipher MA, Chitphakdithai P, Miller JP, Logan BR, King RJ (2009). Adverse events among 2408 unrelated donors of peripheral blood stem cells: results of a prospective trial from the National Marrow Donor Program. Blood.

[3] Schmidt AH, Mengling T, Hernández-Frederick CJ, Rall G, Pingel J (2017). Retrospective analysis of 37,287 observation years after peripheral blood stem cell donation. Biol Blood Marrow Transpl J Am Soc Blood Marrow Transpl.

[4] Platzbecker U, Bornhäuser M, Zimmer K, Lerche L, Rutt C (2005). Second donation of granulocyte-colony-stimulating factor-mobilized peripheral blood progenitor cells: risk factors associated with a low yield of CD34+ cells. Transfusion.

[5] Stroncek DF, Shaw BE, Logan BR, Kiefer DM, Savani BN (2018). Donor experiences of second marrow or peripheral blood stem cell collection mirror the first, but CD34+ yields are less. Biol Blood Marrow Transpl J Am Soc Blood Marrow Transpl.

[6] Tichelli A, Passweg J, Hoffmann T, Gregor M, Kühne T (1999). Repeated peripheral stem cell mobilization in healthy donors: time-dependent changes in mobilization efficiency. Br J Haematol.

[7] La Rubia J, de, Arbona C, Del Cañizo C, Arrieta R, Arriba F de,  (2002). Second mobilization and collection of peripheral blood progenitor cells in healthy donors is associated with lower CD34(+) cell yields. J Hematother Stem Cell Res.

[8] Stroncek DF, Clay ME, Herr G, Smith J, Ilstrup S (1997). Blood counts in healthy donors 1 year after the collection of granulocyte-colony-stimulating factor-mobilized progenitor cells and the results of a second mobilization and collection. Transfusion.

[9] Wang T-F, Wen S-H, Chen R-L, Lu C-J, Zheng Y-J (2008). Factors associated with peripheral blood stem cell yield in volunteer donors mobilized with granulocyte colony-stimulating factors: the impact of donor characteristics and procedural settings. Biol Blood Marrow Transpl J Am Soc Blood Marrow Transpl.

[10] Lenk J, Bornhauser M, Kramer M, Hölig K, Poppe-Thiede K (2013). Sex and body mass index but not CXCL12 801 G/A polymorphism determine the efficacy of hematopoietic cell mobilization: a study in healthy volunteer donors. Biol Blood Marrow Transpl J Am Soc Blood Marrow Transpl.

[11] Schulz M, Karpova D, Spohn G, Damert A, Seifried E (2015). Variant rs1801157 in the 3'UTR of SDF-1ß does not explain variability of healthy-donor G-CSF responsiveness. PLoS ONE.

[12] Garciaz S, Sfumato P, Granata A, Imbert A-M, Fournel C (2020). Analysis of a large single institution cohort of related donors fails to detect a relation between SDF1/CXCR4 or VCAM/VLA4 genetic polymorphisms and the level of hematopoietic progenitor cell mobilization in response to G-CSF. PLoS ONE.

[13] Martín-Antonio B, Carmona M, Falantes J, Gil E, Baez A (2011). Impact of constitutional polymorphisms in VCAM1 and CD44 on CD34+ cell collection yield after administration of granulocyte colony-stimulating factor to healthy donors. Haematologica.

[14] Bräuninger S, Thorausch K, Luxembourg B, Schulz M, Chow KU (2014). Deferrals of volunteer stem cell donors referred for evaluation for matched-unrelated stem cell donation. Bone Marrow Transplant.

[15] Becker P, Schwebig A, Brauninger S, Bialleck H, Luxembourg B (2016). Healthy donor hematopoietic stem cell mobilization with biosimilar granulocyte-colony-stimulating factor: safety, efficacy, and graft performance. Transfusion.

[16] Dauber K, Becker D, Odendahl M, Seifried E, Bonig H (2011). Enumeration of viable CD34(+) cells by flow cytometry in blood, bone marrow and cord blood: results of a study of the novel BD™ stem cell enumeration kit. Cytotherapy.

[17] Hölig K, Kramer M, Kroschinsky F, Bornhäuser M, Mengling T (2009). Safety and efficacy of hematopoietic stem cell collection from mobilized peripheral blood in unrelated volunteers: 12 years of single-center experience in 3928 donors. Blood.

[18] Anderlini P, Przepiorka D, Seong C, Smith TL, Huh YO (1997). Factors affecting mobilization of CD34+ cells in normal donors treated with filgrastim. Transfusion.

[19] Velier M, Granata A, Bramanti S, Calmels B, Furst S (2019). A matched-pair analysis reveals marginally reduced CD34+ cell mobilization on second occasion in 27 related donors who underwent peripheral blood stem cell collection twice at the same institution. Transfusion.

[20] Schmidt H, Schetelig J, Buhrmann K, Kozlova A, Hütter G (2015). Time dependent decrease in efficacy of second G-CSF mobilization in healthy unrelated donors. Blood.

[21] Fiala MA, Park S, Slade M, DiPersio JF, Stockerl-Goldstein KE (2016). Remobilization of hematopoietic stem cells in healthy donors for allogeneic transplantation. Transfusion.

[22] de La Rubia J, Arbona C, de Arriba F, Del Cañizo C, Brunet S (2002). Analysis of factors associated with low peripheral blood progenitor cell collection in normal donors. Transfusion.

[23] Suzuya H, Watanabe T, Nakagawa R, Watanabe H, Okamoto Y (2005). Factors associated with granulocyte colony-stimulating factor-induced peripheral blood stem cell yield in healthy donors. Vox Sang.

[24] Teipel R, Schetelig J, Kramer M, Schmidt H, Schmidt AH (2015). Prediction of hematopoietic stem cell yield after mobilization with granulocyte-colony-stimulating factor in healthy unrelated donors. Transfusion.

[25] Bogunia-Kubik K, Gieryng A, Dlubek D, Lange A (2009). The CXCL12-3'A allele is associated with a higher mobilization yield of CD34 progenitors to the peripheral blood of healthy donors for allogeneic transplantation. Bone Marrow Transpl.

[26] Szmigielska-Kaplon A, Szemraj J, Hamara K, Robak M, Wolska A (2014). Polymorphism of CD44 influences the efficacy of CD34(+) cells mobilization in patients with hematological malignancies. Biol Blood Marrow Transpl J Am Soc Blood Marrow Transpl.

[27] Wong WH, Bhatt S, Trinkaus K, Pusic I, Elliott K, et al. Engraftment of rare, pathogenic donor hematopoietic mutations in unrelated hematopoietic stem cell transplantation. Sci Translational Med. 2020;12(526). 10.1126/scitranslmed.aax6249 PMC7521140 31941826

